# Toxoplasmosis – Awareness and knowledge among medical doctors in Nigeria

**DOI:** 10.1371/journal.pone.0189709

**Published:** 2017-12-19

**Authors:** Akinwale Michael Efunshile, Charles John Elikwu, Pikka Jokelainen

**Affiliations:** 1 Department of Medical Microbiology, Ebonyi State University and Federal Teaching Hospital, Abakaliki, Nigeria; 2 Department of Medical Microbiology & Parasitology, Ben Carson School of Medicine, Babcock University/Babcock University Teaching Hospital, Ilisan-Remo, Ogun State, Nigeria; 3 University of Helsinki, Helsinki, Finland; 4 Estonian University of Life Sciences, Tartu, Estonia; 5 Statens Serum Institut, Copenhagen, Denmark; Scientific Institute of Public Health (WIV-ISP), BELGIUM

## Abstract

*Toxoplasma gondii* is a zoonotic parasite causing high disease burden worldwide. A One Health approach is needed to understand, prevent, and control toxoplasmosis, while knowledge gaps in the One Health aspects have been identified among medical professionals in earlier studies. As a One Health collaboration between veterinary and medical fields, we surveyed the knowledge on toxoplasmosis among medical doctors in Nigeria. The knowledge questions, which the participants answered without consulting literature and colleagues, covered epidemiological One Health aspects as well as clinical interspecialty aspects of *T*. *gondii* infections. Altogether 522 medical doctors from four tertiary hospitals completed the questionnaire. The mean number of correct answers in the knowledge questions was 7.5, and 8.4% of the participants selected at least 12 of the 17 correct answers. The proportion of medical doctors scoring such a high score was significantly higher among those who reported having seen a case of clinical toxoplasmosis than in those who did not. While 62% of the medical doctors participating in our study knew that cats can shed *T*. *gondii* in their feces, 36% incorrectly suggested that humans could do that too. That *T*. *gondii* infection can be meatborne was known by 69%, but that it can be also waterborne only by 28% of the medical doctors participating in our study. Most of the medical doctors, 78%, knew that clinical toxoplasmosis may involve the central nervous system, while only 37% answered that it can involve the eyes. Our results suggested knowledge gaps, which need to be addressed in Continuous Medical Education. The identified gaps included both intersectoral One Health aspects and interspecialty aspects: For prevention and management of toxoplasmosis, knowing the main transmission routes and that the parasite can affect several organs is relevant.

## Introduction

Toxoplasmosis is a neglected zoonotic parasitic disease that is associated with poverty and causes a high disease burden [1]. Toxoplasmosis is caused by the protozoan parasite *Toxoplasma gondii*, which infects a wide range of warm-blooded animals worldwide [2]. It can be transmitted by ingestion of the sporulated oocysts via soil, water, or food contaminated with feces of infected and shedding cats and other felines [3]. Another source of infection is undercooked meat of animals harboring infective tissue cysts [4]. Vertical transmission is a well-known transmission route, and iatrogenic transmission may happen via organ transplant [5, 6, 7].

Nigeria with a population of about 180 million is divided into six geopolitical zones: North-East, North-Central, North-West, South-East, South-West and South-South [8]. There is no control program for toxoplasmosis in any of the zones. The seroprevalence was 78.0% among pregnant women in the South-West zone [9] where epidemiological data suggested contact with feline feces as a major source of infection. Prevalence estimates of 32.6–40.8% were observed among pregnant women in other Nigerian studies [10, 11], and a recent study summarized that the mean prevalence of human *T*. *gondii* infection in Nigeria was 32.0% [12]. Of the more than 350 species of birds and mammals in which *T*. *gondii* infections have been documented [13], few have been investigated in Nigeria. Seroprevalence estimates of 32.6% in domestic cats [14] and 15.4% in dogs [15], 13.9% in cattle and 29.1% in pigs [16], and 6.7% in sheep and 4.6% in goats [17] have been reported.

Most *T*. *gondii* infections in immunocompetent individuals of most host species, including humans, are subclinical. The symptoms in humans may include fever and lymphadenopathy, but the infection can lead to severe clinical manifestations and mortality, especially in the immunocompromised and in children with congenital infection [7, 18]. The diagnosis of *T*. *gondii* infection and toxoplasmosis is usually serological, and molecular methods are used for direct detection of the parasite [19]. Currently, there is no curative treatment, and immunocompetent patients usually do not need treatment.

Because of the zoonotic nature of *T*. *gondii*, a One Health approach is needed to understand, prevent, and control toxoplasmosis [20]. However, for example, among medical doctors and nurses who provide prenatal care in Brazil [21], knowledge gaps in One Health aspects were evident: despite most of the participants knew that cats can shed the parasite in their feces, more than half of the participants wrongly suggested that dogs could shed as well. We designed our current study as a One Health collaboration between veterinary and medical fields [22], and surveyed the knowledge on toxoplasmosis among medical doctors in Nigeria to identify possible knowledge gaps and need for Continuous Medical Education (CME).

## Materials and methods

### Ethical statement

The study was approved by the Ethics and Research Committee of the Federal Teaching Hospital, Abakaliki. Participation was voluntary and anonymous, and no personal data were collected. By filling in the questionnaire, the participants gave consent to use the answers for research purposes. This was explicitly written in the questionnaire (S1 File). A signed written consent was not collected to ensure anonymity of the participants. The results are reported so that individual participants cannot be identified.

### Study design and setting

We designed a cross-sectional questionnaire-based study that was carried out in four tertiary hospitals in Nigeria. The hospital selection was a convenience selection. Two hospitals were chosen from the South-East geopolitical zone of Nigeria where previous studies on toxoplasmosis were scanty, and two hospitals were chosen from the South-West zone where previous studies had shown high *T*. *gondii* seroprevalence [12]. Two of the hospitals were located in Ogun state, one in Ebonyi state, and one in Anambra state.

The sample was a convenience sample. We aimed for a sample size of at least 400 participants, based on sample size calculations using OpenEpi software [23]. With expected 10–15% to be classified having high knowledge score, a minimum of 98–138 participants from each hospital were needed for an estimate of the proportion at hospital level at 90% confidence level, and a minimum of 390–552 participants altogether were needed for an overall estimate at 99.9% confidence level. This was evaluated as sufficient to detect differences of relevant magnitude (calculated using 70% and 90%) between two groups, with a power of 80% and two-sided confidence level of 95%.

Anonymous, self-completed questionnaires were used to collect basic background information of the participants and to survey their knowledge on toxoplasmosis using questions on its epidemiology and clinical features, perception of knowing how and when to treat toxoplasmosis, and history of having seen a case of clinical toxoplasmosis. The participants were all medical doctors. Their specialties were not asked to ensure their anonymity, only the overall length of their experience in the medical field was asked.

The questionnaire was a standardized written questionnaire, i.e. administered to all participants in identical format. It was in English, and distributed as printed on a single A4-paper (S1 File). The participants were instructed to answer based on their current knowledge and opinions, without any external information sources. Author(s) were present to ensure no external information sources were used. The participants answered by circling the answers from the options given, and selecting several answers per question was allowed.

### Statistical analyses

The 95% confidence intervals (CI) were calculated and preliminary 2-by-2-table comparisons were done using OpenEpi software [23]. Further analyses were performed using Stata IC 13.1 (2013) software (Stata Corporation, TX, USA). *P* values <0.05 were considered statistically significant.

The logistic regression analyses included gender (dichotomous: female vs. male), length of professional experience (categorized: <5 years, 5–10 years, or >10 years), reportedly knowing how and when to treat toxoplasmosis (dichotomous: “Yes” vs. “No”; “I am not sure” regarded as no data), reportedly having seen a case of clinical toxoplasmosis (dichotomous: “Yes” vs. “No”; “I am not sure” regarded as no data), and scoring a high knowledge score (dichotomous: selecting at least 12 of the 17 correct answers vs. selecting fewer correct answers). The cross-sectional study design did not allow evaluation of the variables on a timeline, and thus model building was done for predicting scoring a high knowledge score and for predicting having seen a case of clinical toxoplasmosis. Multivariable models were built by including all variables with a liberal *P* value of <0.2 followed by removal of variables that were not statistically significant and did not act as confounders. The predictive ability of the models was evaluated as area under the receiver operating characteristic (ROC) curve, which is both a numerical and a visual tool.

## Results

Altogether 522 medical doctors completed the questionnaire (Table 1, S2 File). Of the participants, 33.1% were female and 65.5% were male. Almost half (48.1%) of the participants had 5–10 years of experience in the medical field, while 27.8% reported shorter and 23.2% longer experience. Some participants did not answer all questions; e.g. gender was unknown for seven participants and length of professional experience for five.

**Table 1:** Proportion of medical doctors scoring a high knowledge score in the questionnaire surveying knowledge on toxoplasmosis in Nigeria, by their background information.

	N	n with high score	% with high score	95% confidence interval
Hospital 1 (South-East zone)	164	9	5.49	2.71–9.83
Hospital 2 (South-East zone)	177	19	10.73	6.78–15.96
Hospital 3 (South-West zone)	61	9	14.75	7.44–25.35
Hospital 4 (South-West zone)	117	7	5.98	2.65–11.47
Female	173	10	5.78	2.97–10.06
Male	342	34	9.94	7.10–13.46
Experience <5 years	144	12	8.33	4.59–13.74
Experience 5–10 years	252	21	8.33	5.37–12.25
Experience >10 years	121	11	9.09	4.87–15.25
Reportedly knows how and when to treat toxoplasmosis	165	18	10.91	6.80–16.38
Reportedly does not know how and when to treat toxoplasmosis	181	16	8.84	5.32–13.67
Has seen a case of clinical toxoplasmosis	75	13	17.33	10.00–27.15
Has not seen a case of clinical toxoplasmosis	367	27	7.36	5.00–10.38
All	522	44	8.43	6.27–11.05

The mean number of correct answers in the knowledge questions was 7.5 (range 0–16), and 44 (8.4%) of the participants scored a high score (Tables 1 and 2). Uncertainty was commonly expressed by the participants. For example, 177 (33.9%) replied that they were not sure whether they know how and when to treat toxoplasmosis, and 78 (14.9%) replied that they were not sure whether they had seen a case of clinical toxoplasmosis. The “not sure” option was also commonly selected for the knowledge questions (Table 2).

**Table 2:** Distribution of answers of medical doctors to the knowledge questions on toxoplasmosis. The seventeen answers that were considered correct are highlighted with bold font.

	n selecting this answer	% selecting this answer (N = 522)	95% confidence interval
*Toxoplasma gondii* is a bacterium	29	5.56	3.82–7.78
*Toxoplasma gondii* is a virus	15	2.87	1.68–4.59
***Toxoplasma gondii* is a parasite**	419	80.27	76.69–83.51
*Toxoplasma gondii* is a fungi	27	5.17	3.51–7.34
*Toxoplasma gondii* is an insect	0	0.00	0.00–0.57
Not sure what *T*. *gondii* is	20	3.83	2.42–5.75
***Toxoplasma gondii* can infect humans**	419	80.27	76.69–83.51
***Toxoplasma gondii* can infect pigs**	97	18.58	15.42–22.09
***Toxoplasma gondii* can infect sheep**	69	13.22	10.51–16.33
***Toxoplasma gondii* can infect camels**	28	5.36	3.66–7.56
***Toxoplasma gondii* can infect dogs**	115	22.03	18.63–25.74
***Toxoplasma gondii* can infect cats**	298	57.09	52.81–61.29
*Toxoplasma gondii* can infect fish	4	0.77	0.24–1.84
***Toxoplasma gondii* can infect chicken**	7	1.34	0.59–2.64
*Toxoplasma gondii* can infect spiders	0	0.00	0.00–0.57
Not sure what *T*. *gondii* can infect	21	4.02	2.57–5.98
*Toxoplasma gondii* can be shed in the feces of humans	186	35.63	31.61–39.81
*Toxoplasma gondii* can be shed in the feces of pigs	56	10.73	8.29–13.61
*Toxoplasma gondii* can be shed in the feces of sheep	44	8.43	6.27–11.05
*Toxoplasma gondii* can be shed in the feces of camels	11	2.11	1.11–3.63
*Toxoplasma gondii* can be shed in the feces of dogs	108	20.69	17.38–24.33
***Toxoplasma gondii* can be shed in the feces of cats**	324	62.07	57.84–66.16
*Toxoplasma gondii* can be shed in the feces of fish	4	0.77	0.24–1.84
*Toxoplasma gondii* can be shed in the feces of chicken	2	0.38	0.06–1.26
*Toxoplasma gondii* can be shed in the feces of spiders	0	0.00	0.00–0.57
Not sure what can shed *T*. *gondii* in the feces	65	12.45	9.82–15.50
***Toxoplasma gondii* infection can be meatborne**	361	69.16	65.09–73.01
*Toxoplasma gondii* infection cannot be meatborne	46	8.81	6.60–11.48
Not sure whether *T*. *gondii* infection can be meatborne	105	20.11	16.84–23.72
***Toxoplasma gondii* infection can be waterborne**	146	27.97	24.24–31.94
*Toxoplasma gondii* infection cannot be waterborne	178	34.10	30.13–38.25
Not sure whether *T*. *gondii* infection can be waterborne	190	36.40	32.35–40.60
**Clinical toxoplasmosis may involve: central nervous system**	407	77.97	74.26–81.37
**Clinical toxoplasmosis may involve: fetus**	227	43.49	39.28–47.77
Clinical toxoplasmosis may involve: Red blood cells	37	7.09	5.11–9.54
**Clinical toxoplasmosis may involve: Lungs**	116	22.22	18.81–25.94
**Clinical toxoplasmosis may involve: Eyes**	193	36.97	32.91–41.18
Not sure what clinical toxoplasmosis may involve	48	9.20	6.94–11.91
**Most *T*. *gondii* infections are subclinical**	365	69.92	65.88–73.74
Most *T*. *gondii* infections are not subclinical	48	9.20	6.94–11.91
Not sure whether most *T*. *gondii* infections are subclinical	102	19.54	16.31–23.11
***Toxoplasma gondii* can cause blindness**	320	61.30	57.07–65.41
*Toxoplasma gondii* cannot cause blindness	43	8.24	6.10–10.84
Not sure whether *T*. *gondii* can cause blindness	157	30.08	26.26–34.12

The proportion of participants scoring a high score was significantly higher (2-tailed Mid-*P* exact *P* <0.05) among those who had reportedly seen a case of clinical toxoplasmosis than in those who had not. A medical doctor who had seen a case of clinical toxoplasmosis had 2.6 (95% CI 1.3–5.4) times higher odds to score a high knowledge score than a medical doctor who did not report seeing one. This univariable model used the data from 442 medical doctors and had low predictive power (area under ROC curve 0.58).

Overall, 17% of the medical doctors who participated in our study reported they had seen a case of clinical toxoplasmosis (Table 1). The proportion was the same, 17%, in the South-East zone and in the South-West zone. The final multivariable model for predicting having seen a case of clinical toxoplasmosis used the data from 324 medical doctors and had three variables: length of professional experience, scoring high knowledge score, and reportedly knowing how and when to treat toxoplasmosis (Table 3). The predictive power of this model was good (area under ROC curve 0.77, the curve extended reasonably well into the upper left corner of the graph).

**Table 3:** Multivariable logistic regression model for predicting having seen a case of clinical toxoplasmosis among medical doctors in Nigeria.

	Odds ratio	95% confidence interval	*P* value
Experience <5 years	Reference		
Experience 5–10 years	2.6	1.1–6.5	0.036
Experience >10 years	3.3	1.3–8.8	0.016
Lower knowledge score	Reference		
High knowledge score	2.9	1.2–7.3	0.019
Reportedly not knowing how and when to treat toxoplasmosis	Reference		
Reportedly knowing how and when to treat toxoplasmosis	6.7	3.3–13.7	0.000

## Discussion

Sub-Saharan Africa faces great health management challenges: it bears 25% of global health burden, while it has only 3% of the global health workforce [24]. In Nigeria, the health workforce is mainly concentrated in the urban tertiary health care services [25], which our study focused on. They are key units in the health care and their role in educating the profession is paramount.

The strengths of our study include the good sample size and multi-hospital study design. Due to limited resources, we were unable to cover all six geopolitical zones, and the results may thus not represent all the zones. Interestingly, there was no obvious difference between the results from the South-East geopolitical zone where previous studies on toxoplasmosis were scanty, and from the South-West zone where previous studies showed high *T*. *gondii* seroprevalence (Table 1) [12]. The study was planned carefully and the questionnaire was a standardized written questionnaire, administered to all participants in identical format, which increases its reliability. The low-cost study design was successful, and the multi-hospital collaboration as well as the international collaboration between the veterinary and medical fields were exemplary and encouraging. The major limitations of our study arose from the cross-sectional study design, convenience sample, and limited background information of the participants. Moreover, validity of the perception question was not evaluated, and we had no time limit for the question about having seen a case.

We defined high knowledge score as selecting at least 12 of the 17 correct answers (Table 2). This was reached by less than a tenth of the medical doctors participating in our study. What score could be seen as good or adequate was not defined in this study–some of the knowledge questions may be considered trivial, while the baseline knowledge and awareness of the One Health aspects and numerous possible clinical manifestations are important. It should be emphasized that the participants answered without using any external information sources, while consulting literature and colleagues are normal practices in medical profession. The questionnaire perhaps made the participants reflect on their knowledge and select “not sure” fairly easily (Table 2). Uncertainty was also evident in the self-reporting of knowing how and when to treat toxoplasmosis (Table 1). It should be emphasized that the question measured self-reported perception, and it remains unknown whether the 32% answering they knew how and when to treat toxoplasmosis actually did, as well as whether the 35% answering they did not know referred to the wide range of clinical manifestations.

Some aspects about toxoplasmosis appeared better known, for example, 62% of the medical doctors participating in our study knew that cats can shed *T*. *gondii* in their feces, 69% knew that *T*. *gondii* infection can be meatborne, and 78% knew that clinical toxoplasmosis may involve the central nervous system (Table 2). However, several knowledge gaps were identified, as, for example, more than a third of the medical doctors participating in our study wrongly suggested that humans could shed *T*. *gondii* in their feces, a small proportion knew the parasite can infect chicken, less than a third knew *T*. *gondii* infection can be waterborne, and only a bit more than a third knew ocular involvement is possible (Table 2). For management of toxoplasmosis and for providing efficient advice regarding preventing it, knowing that the parasite can affect several organs and understanding the main transmission routes are relevant. The knowledge gaps we identified can be addressed in Continuous Medical Education (CME), and interspecialty and intersectoral collaboration could help in widening the discussion to cover the different aspects and One Health thinking.

The topic, *T*. *gondii* -related knowledge among healthcare professionals, has been of research interest in several countries [21, 26–30], however the studies are not easily comparable due to different approaches and focuses. Comparing our findings with the results of the study among 118 health care workers in Brazil [21] as well as with the results of another similar study among 100 medical doctors in Mexico [26] suggested that the epidemiological aspects of toxoplasmosis were better known in Brazil and Mexico than in Nigeria: for example, 95.8% (95% CI not provided) in Brazil [21] and 89.0% (95% CI not provided) in Mexico [26] knew that *T*. *gondii* can be shed by cats, whereas a significantly lower proportion of medical doctors in Nigeria, 62.07% (95% CI 57.84–66.16), indicated knowing this by selecting the correct answer (2-tailed Mid-*P* exact *P* <0.001 for both comparisons). The majority of the participants in all three studies indicated knowing that *T*. *gondii* infection is mostly subclinical: 73.9% (95% CI not provided) in Brazil [21], 59.0% (95% CI not provided) in Mexico [26], and 69.92% (95% CI 65.88–73.74) in our study in Nigeria. These three proportions are not directly comparable due to differently formulated questions.

The logistic regression models suggested association between having seen a case of clinical toxoplasmosis and scoring a high knowledge score, but the data did not allow placing these on a timeline. Moreover, whether this association could be partly explained by specialties of the participating medical doctors could not be evaluated in this study. We can only speculate around the association, in both possible directions, more generally: it is possible that having seen a case encouraged reading and learning about the parasite, as well as that knowing the parasite well helped to keep it on the list of differential diagnoses and thus made it more likely to detect a case. While the incidence of clinical toxoplasmosis will hopefully decline in the future decades, case-based CME could be emphasized.

## Conclusions

While many aspects of *T*. *gondii* were known by the majority of the medical doctors participating in our study, our results suggested knowledge gaps in intersectoral One Health aspects and interspecialty aspects of the zoonotic parasite. These knowledge gaps should be addressed in CME and by intersectoral and interspecialty collaboration. The observed association between having seen a case of clinical toxoplasmosis and scoring a high knowledge score encourages using a case-based approach in CME.

## Supporting information

S1 File The questionnaire to survey knowledge on toxoplasmosis among medical doctors. (PDF)

S2 File Dataset. (XLSX)
