# Supplementary material for: Toxoplasmosis – Awareness and knowledge among medical doctors in Nigeria
Source: PLoS One. 2017 Dec 19;12(12):e0189709. doi: 10.1371/journal.pone.0189709 (PMC5736225; doi:10.1371/journal.pone.0189709)
Supplement: S1 File — (PDF) [file pone.0189709.s001.pdf]

Dear colleague,

This questionnaire for medical doctors is part of a study on T*oxoplasma gondii* in Nigeria. We kindly ask you for your time and sincere opinions: please fill in this questionnaire based on your current knowledge, without using any external information sources. By doing so, you give permission to use the answers for research purposes.

We plan to include a summary of the results in an international research publication. The results will be reported so that an individual participant cannot be recognized. The new information gathered will also help to evaluate the needs for continued professional education.

Please circle your answers from the options given. You may select several answers for each question.

Sex	Female	Male	
Years of experience in the medical field	< 5	5-10	>10
Toxoplasma gondii is a	Bacterium<br>Fungi	Virus<br>Insect	Parasite<br>I am not sure
T. gondii can infect	Humans<br>Camels<br>Fish	Pigs<br>Dogs<br>Chicken	Sheep<br>Cats<br>Spiders<br>I am not sure
T. gondii can be shed in the feces of	Humans<br>Camels<br>Fish	Pigs<br>Dogs<br>Chicken	Sheep<br>Cats<br>Spiders<br>I am not sure
T. gondii infections can be meatborne	Yes	No	I am not sure
T. gondii infections can be waterborne	Yes	No	I am not sure
Clinical toxoplasmosis may involve	CNS<br>Lungs	Fetus<br>Eyes	Red blood cells<br>I am not sure
Most *T. gondii* infections are subclinical	Yes	No	I am not sure
T. gondii can cause blindness	Yes	No	I am not sure
I know how and when to treat toxoplasmosis	Yes	No	I am not sure
I have seen a case of clinical toxoplasmosis	Yes	No	I am not sure

Please return this sheet back to our designated staff. **Thank you.**

For enquiries, please do not hesitate to contact us: Dr Efunshile AM-drefunshile@yahoo.com
